# Rhizosphere Soil Fungal Communities of Aluminum-Tolerant and -Sensitive Soybean Genotypes Respond Differently to Aluminum Stress in an Acid Soil

**DOI:** 10.3389/fmicb.2020.01177

**Published:** 2020-05-28

**Authors:** Qihan Shi, Yuantai Liu, Aoqing Shi, Zhandong Cai, Hai Nian, Martin Hartmann, Tengxiang Lian

**Affiliations:** ^1^The State Key Laboratory for Conservation and Utilization of Subtropical Agro-bioresources, South China Agricultural University, Guangzhou, Guangdong, China; ^2^The Key Laboratory of Plant Molecular Breeding of Guangdong Province, College of Agriculture, South China Agricultural University, Guangzhou, Guangdong, China; ^3^Institute of Agricultural Sciences, ETH Zurich, Zurich, Switzerland

**Keywords:** rhizosphere fungal community, aluminum toxicity, soybean genotypes, metabarcoding, network

## Abstract

Different soybean genotypes can differ in their tolerance toward aluminum stress depending on their rhizosphere-inhabiting microorganisms. However, there is limited understanding of the response of fungal communities to different aluminum concentrations across different genotypes. Here, we used metabarcoding of fungal ribosomal markers to assess the effects of aluminum stress on the rhizosphere fungal community of aluminum-tolerant and aluminum-sensitive soybean genotypes. Shifts in fungal community structure were related to changes in plant biomass, fungal abundance and soil chemical properties. Aluminum stress increased the difference in fungal community structure between tolerant and sensitive genotypes. *Penicillium*, *Cladosporium* and *Talaromyces* increased with increasing aluminum concentration. These taxa associated with the aluminum-tolerant genotypes were enriched at the highest aluminum concentration. Moreover, complexity of the co-occurrence network associated with the tolerant genotypes increased at the highest aluminum concentration. Collectively, increasing aluminum concentrations magnified the differences in fungal community structure between the two studied tolerant and sensitive soybean genotypes. This study highlights the possibility to focus on rhizosphere fungal communities as potential breeding target to produce crops that are more tolerant toward heavy metal stress or toxicity in general.

## Introduction

Aluminum (Al) toxicity is one of the most widespread problem in acidic soils, affecting approximately 40% of the arable land worldwide (Ma et al., 2001; Pierluigi et al., 2008). In acidic soils with pH values below five, insoluble forms of Al are turned into soluble Al^3+^ ions (Kinraide, 1991; Da Mota et al., 2008). Many studies have reported that Al^3+^ with high phytotoxicity causes inhibition of nitrate reductase activity and disruption of nitrogen reduction and assimilation (Zhao and Shen, 2018). Moreover, the absorption and utilization of other soil elements such as phosphorus, potassium and iron by plant roots are also affected by Al stress (Pfeffer et al., 1986; Delhaize and Ryan, 1995; Pineros and Kochian, 2001). Therefore, increased concentration of soluble Al can lead to inhibition of plant root growth and, thus, reduction in crop yield (Kochian, 1995; Kong et al., 1997; Exley, 2012; Riaz et al., 2018).

There have been several reports on possible mechanisms of plants to increase tolerance toward high Al^3+^ concentrations, among which the chelation of Al^3+^ through organic acids such as malic acid, oxalic acid, or citric acid excreted by plant roots is considered to be one of the vital mechanisms (Ma et al., 2001). The different levels of Al tolerance vary significantly among genotypes, largely because of different types and quantities of secreted organic acids (Miyasaka et al., 1991; Wu et al., 2018). For instance, more organic acids are excreted by Al-tolerant (Al-T) soybean genotypes when compared with Al-sensitive (Al-S) genotypes, leading to chelation of more Al^3+^ (Yang et al., 2010). However, the increased amounts of organic acids not only regulate Al^3+^ concentrations in soil (Silva et al., 2004), but also shapes the microbial community composition at the root-soil interface through providing nutrients (Jones et al., 1996; Bürgmann et al., 2005).

Microorganisms greatly contribute to plant health and productivity (Mendes et al., 2013; Li et al., 2014a, b). When subjected to environmental stress, plants have the potential to recruit specific microbes to the root system to alleviate the stress (Rodriguez et al., 2019). For example, some plant-growth-promoting bacteria (PGPB) in the soil such as *Klebsiella*, *Serratia*, and *Enterobacter* have the capacity to form Al^3+^-siderophore complexes and improve P-uptake efficiency to cope with Al stress (Mora et al., 2017). Previous studies have investigated the structure of rhizosphere bacterial communities in Al-T and Al-S plants and suggested that Al-T genotypes recruit certain bacterial species that help mitigating Al toxicity (Yang et al., 2012a; Wang et al., 2013; Lian et al., 2019). However, these studies have focused on rhizosphere bacteria as key players, ignoring that fungal species play important roles in nutrient cycling and stress tolerance (Kawai et al., 2000; Peltoniemi et al., 2012). For instance, some fungi, such as *Penicillium* and *Aspergillus* have been shown to improve Al-tolerance by producing organic acids and at the same time provisioning plants root with nitrogen and phosphorus to promote growth and increase vitality (Kiers et al., 2011).

In this study, differences in rhizosphere fungal community structure of cultivated soybean genotypes with different tolerance levels to Al were assessed using high-throughput DNA sequencing of the internal transcribed spacer (ITS) region, and correlated with plant growth and chemical soil properties. Based on the higher adaptability of Al-tolerant soybean genotypes to Al toxicity, we hypothesized that (1) fungal diversity of Al-T genotypes is higher when compared to Al-S genotypes, and (2) the response of fungal community structure to aluminum between Al-T and Al-S soybean genotypes is different, and these differences increase with increasing Al concentration.

## Materials and Methods

### Soil Source and Plant Materials

The soil used in the pot experiment was collected from an agricultural field in Suixi County (110°25′N, 21°32′E), Guangdong Province, China, in June 2017. The chemical properties are provided in Supplementary Table S1. The soybean (*Glycine max* L.) cultivars used in this study included the Al-tolerant genotypes HuaChun2 and Lee as well as the Al-sensitive genotypes LiuDou1 and Young (Hanson and Kamprath, 1979; Hanson, 1991; Wen, 2007).

### Experimental Description and Rhizosphere Soil Collection

A completely randomized block design was set up in a greenhouse of the South China Agricultural University in Guangzhou, China. Before the experiment, the soil was air-dried and sieved with a 4 mm mash size. Soybeans were seeded into 2 kg soil per pot (150 mm height × 200 mm top diameter and 150 mm bottom diameter). Six seeds of equal size were planted in each pot and germinating soybeans were subsequently removed to obtain two plants per pot after eight days of growth. Aluminum sulfate Al_2_(SO_4_)_3_ was used as Al source. Three different Al concentrations ranging from 0 (none) to 0.2 (low) and 0.4 (high) g Al^3+^ kg^–1^ soil were applied as treatments. Simultaneously, the same levels of Al concentrations were also applied without planting of soybean (no plant controls, CK). Each treatment in the experiment was installed in three replications. The experimental conditions of daytime temperatures ranged from 28 to 32°C, and night time temperatures ranged from 16 to 20°C. Soil moisture was kept at 80% of the moisture level in the field by weighting and watering.

Because Al toxicity is affecting the flowering stages, six rhizosphere soil samples (three replications per cultivar) at 0 (zero), 0.2 (low) and 0.4 (high) g kg^–1^ Al^3+^ concentrations were collected at 40, 50, and 65 days after planting, which represented the flowering stages at the three different Al concentrations, respectively. All rhizosphere soil samples were collected by gently shaking the plant root to remove loosely attached soil, and then the soil adhering to the root system was transported to an aseptic bag filled with 30 ml of phosphate-buffered saline and processed for molecular microbial community analysis as previously described (Shi et al., 2015). Two grams of rhizosphere soil was taken from each sample, placed in a sterilized microcentrifuge tube and stored at −80°C for DNA extraction. The remaining soil sample was stored at 4°C until measuring the chemical properties.

### Soil Properties Measurement

Soil pH was measured in aqueous solution using a FE20-FiveEasy^TM^ pH meter (Mettler Toledo, Columbus, United States). Total carbon (TC) and nitrogen (TN) were determined by a vario MAX CN Elemental Analyser (Elementar Analysensysteme, Hanau, Germany). Total soil potassium (TK) was measured by inductively coupled plasma-atomic emission spectrometry on an ICPS-7500 (Shimadzu, Kyoto, Japan) (Lian et al., 2019). Total soil phosphorus (TP) was determined by digesting with H_2_SO_4_-HClO_4_ as previously described (Huang et al., 2011) and measured by a continuous flow analytical system (Skalar, Breda, Netherlands). The titrimetric method was used to evaluate soil exchangeable H^+^ and Al^3+^ (Abreu et al., 2003). Soil nitrate (NO_3_^–^-N) and ammonium (NH_4_^+^-N) were extracted with 2 mol L^–1^ KCl, and then assayed by a continuous flow analytical system (Skalar, Breda, Netherlands) as previously described (Jiang et al., 2017). Soil available phosphorus (AP) was determined by molybdenum-antimony colorimetric method (Sun et al., 2015).

### Molecular Genetic Analyses

Rhizosphere soil DNA was extracted using the Fast DNA SPIN Kit for Soil (MP Biomedicals, Santa Ana, United States) as specified in the manufacturer’s instructions. The internal transcribed spacer region ITS1 of the fungal ribosomal operon was amplified using primer ITS1 (CTTGGTCATTTAGAGGAAGTAA; Gardes and Bruns, 1993) and ITS2 (GCTGCGTTCTTCATCGATGC; White et al., 1990) with a unique six nt barcode at the 5′ end. PCR amplification for sequencing was carried out in a volume of 30 μL with 15 μL of Phusion High-Fidelity PCR Master Mix (New England Biolabs, Beverly, MA, United States), 0.2 μM of forward and reverse primers, and 10 ng template DNA. PCR reaction cycling conditions were 1 × (60 s, 98°C), 30 × (10 s, 98°C; 30 s, 50°C; 60 s, 72°C), and a final elongation cycle at 72°C for 5 min. Quantitative PCR (qPCR) was done using the same primers and according to the protocol described previously (Yao et al., 2017). Briefly, each PCR reaction contained 10 μL of SYBR Premix Ex Taq^TM^ (Takara, Dalian, China), 1.0 μL of 10 mM forward and reverse primers, 1.0 μL of soil DNA, and 7.0 μL of sterilized water. qPCR was performed in an ABI 7900 system following a program that started with initial denaturation at 95°C for 45 s, followed by 32 cycles of 95°C for 15 s, 58°C for 20 s, 72°C for 20 s, and one final cycle of 45°C for 10 min for cooling. Sequencing libraries were generated from PCR products using NEB Next Ultra^TM^DNA Library Prep Kit for Illumina (New England Biolabs, Ipswich, MA, United States) according to the manufacturer’s protocol. Libraries were paired-end sequenced on an Illumina MiSeq platform using 2 × 300 bp chemistry (Illumina Inc., San Diego, CA, United States). Raw sequences were deposited in the NCBI short-read archive under the accession numbers PRJNA561469.

### Bioinformatic Processing

Raw sequence data were processed using QIIME v1.19.1 (Caporaso et al., 2010). In brief, reads shorter than 200 bp and average quality score below 20 were removed, and paired-end reads were merged into full-length amplicon sequences with FLASH (Magoč and Salzberg, 2011). Potentially chimeric sequences were detected by running the UCHIME algorithm (Edgar et al., 2011). The CD-HIT program was used to cluster OTUs at 97% sequence identity (Li and Godzik, 2006). Taxonomic assignments of OTU representative sequences was done against the UNITE database^1^ (Abarenkov et al., 2010) using the RDP naïve Bayesian classifier (Wang et al., 2007). All the samples were randomly resampled to the same sequence depth (29,169 sequences per sample), in order to reduce the influence of sequencing depth on treatment effects. In addition, we filtered the OTU table to remove rare OTUs (less than 0.001% abundance), sparse OTUs (OTUs that did not occur in at least three samples), and outlier OTUs defined as OTUs that showed a ratio of more than 10:1 from the highest to the second highest value.

### Statistics Analyses

Alpha-diversity metrics, i.e., Chao1 richness estimator and Shannon diversity index, were calculated with the QIIME software. Non-metric multidimensional scaling (NMDS; Kruskal, 1964) based on Bray-Curtis dissimilarities, non-parametric multivariate analysis of variance (PERMANOVA; Anderson, 2001), canonical correspondence analysis (CCA; Braak, 1986), and Mantel test (Mantel, 1967) were conducted in R (R Development Core Team, 2006) using the functions *metaMDS*, *adonis*, *cca*, and *mantel* of the “vegan” package (Oksanen et al., 2013). Canonical analysis of principal coordinates (CAP; Anderson and Willis, 2003) was performed using the CAPdiscrim function in the R package “BiodiversityR” (Kindt and Coe, 2005). The relative abundance of fungal phyla was visualized by the R package “circlize” (Gu et al., 2014). Differences between treatments in soil properties and the relative abundances of fungal genera being associated with Al-T genotypes were assessed with two-way analysis of variance (ANOVA) in Genstat (Version 13.0), followed by Duncan’s multiple range test (*P* < 0.05).

The association strength of each OTU and higher level taxa with a particular genotype × Al-concentration group or group combination was determined using correlation-based indicator species analysis (De Cáceres and Legendre, 2009) with all possible site combinations (De Cáceres et al., 2010) using the multipatt function in the R package indicspecies (De Cáceres and Legendre, 2009). *P*-value correction for multiple testing was performed using the false discovery rate correction according to Storey (2002) using the R package qvalue (Storey et al., 2015). A bipartite association network was generated based on the indicator results, which were the taxa that were identified in the indicator species analysis, to visualize positive associations of particular OTUs with specific treatments or treatment combinations as described previously (Hartmann et al., 2015) using the Allegro Fruchterman-Reingold algorithm in CYTOSCAPE 3.8 (Shannon et al., 2003).

Co-occurrence networks were utilized to assess the relationships between fungal OTUs with a relative abundance >0.1%. Pairwise correlations between OTUs were obtained by calculating Spearman correlation coefficients using the R package “psych” (Revelle, 2017), and correlations with *r* > 0.8 and *P* < 0.05 were included in the network. Co-occurrence networks visualization were constructed for each plant genotype and Al treatment using Gephi v.0.9.2 (Bastian et al., 2009). Topological properties of the networks were calculated to elucidate community structure differences across genotypes and Al treatments. Hubs were defined as OTUs in the network that show high degree, high betweenness centrality and high closeness centrality (Berry and Widder, 2014; Agler et al., 2016).

## Results

### Soybean Biomass, Soil Fungal Abundance and Diversity

**FIGURE 1:**
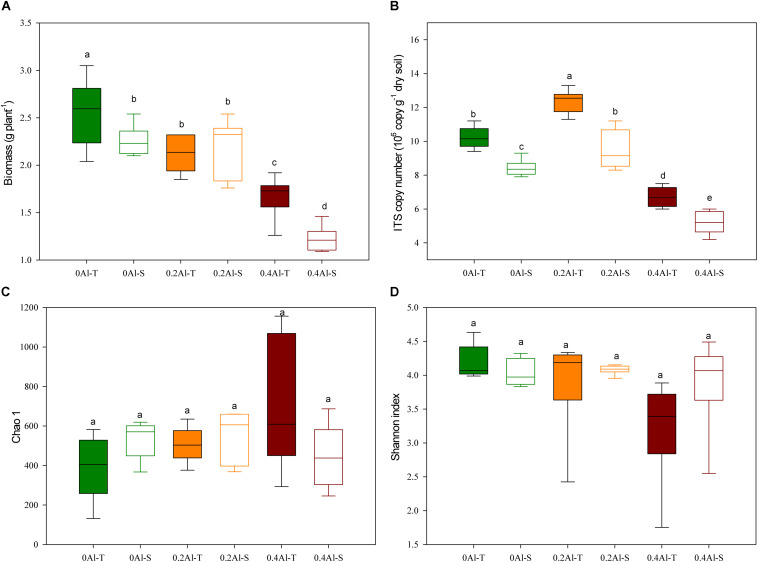
Effects of Al addition (0 g kg^–1^, 0.2 g kg^–1^ and 0.4 g kg^–1^ Al^3+^) on soybean biomass **(A)**, abundance of soybean rhizosphere fungal ribosomal ITS1 copies **(B)**, soybean rhizosphere soil fungal Chao1 estimated richness **(C)** and fungal Shannon diversity index **(D)**. One-way ANOVA with Student’s *t*-test showed significant differences between the Al-T and Al-S (*P* < 0.05). Error bars on data points represent the standard error of the mean (*n* = 6). Al-T: Al-tolerant soybean genotypes; Al-S: Al-sensitive soybean genotypes.

The biomass of all soybeans decreased with Al concentration, but significant differences between tolerant (Al-T) and sensitive (Al-S) genotypes only occurred at the highest Al concentration (Figure 1A). Fungal abundance varied from 4.2 to 13.3 × 10^7^ ITS1 copies g^–1^ dry soil, showing a significant increase at the low Al concentration and a significant decrease at the high Al concentration when compared to the control (Figure 1B, *P* < 0.001). At all Al concentrations, Al-T genotypes had significant higher fungal abundance than Al-S genotypes (Figure 1B). There were no significant differences in fungal alpha diversity (e.g., Chao1 richness and Shannon diversity index) across the different Al concentrations and between the sensitive and tolerant genotypes (Figures 1C,D).

### Soil Chemical Properties

The chemical properties of all soil samples are shown in Supplementary Table S1. Exchangeable Al^3+^ and H^+^ significantly increased and pH decreased with increasing Al concentration (*P* < 0.05). At the highest Al concentration, exchangeable Al^3+^ was significantly higher in pots with Al-S compared to Al-T genotypes. Available phosphorus, NO_3_^–^-N and NH_4_^+^-N were significantly lower in pots with Al-S genotypes at high Al concentration.

### Rhizosphere Fungal Community Structure

**FIGURE 2:**
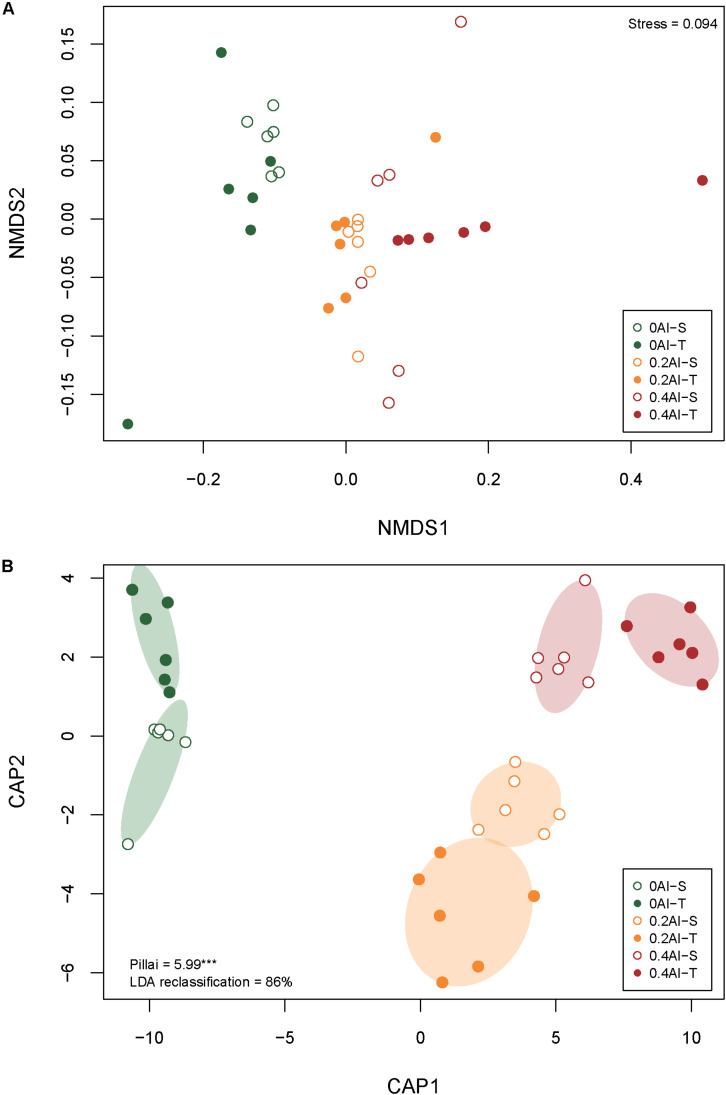
Non-metric multidimensional scale (NMDS) **(A)** and canonical analysis of principal coordinates (CAP) **(B)** based on Bray-Curtis dissimilarities showing differences in rhizosphere fungal community structures at 0 g kg^–1^ 0.2 g kg^–1^, and 0.4 g kg^–1^ Al concentrations The stress value for the NMDS as well as Pillai’s trace and the leave-one-out re-allocation success rate of the linear discriminant analysis for the CAP are provided in the plot corners. Al-T: Al-tolerant soybean genotypes; Al-S: Al-sensitive soybean genotypes.

A total of 2,019,383 quality-filtered fungal ITS1 sequences were obtained. Number of reads ranged from 29,169 to 59,719 with a mean read count of 44,875 ± 9139. Fungal communities showed distinct compositions between rhizosphere and the unplanted soil (Supplementary Figure S1). The rhizosphere fungal communities changed with increasing Al concentration (Figure 2A and Table 1). Sensitive and tolerant genotypes harbored significantly different communities at all treatment levels, but separation was strongest at the high Al concentration (Figure 2B and Table 1).

**TABLE 1:** Effects of aluminum concentration and soybean genotypes on fungal community structure assessed by permutational multivariate analysis of variance (PERMANOVA).

**Factor**	**F**	**R^2^**	***P***
Al	10.5	0.325	0.0001
trait^a^	2	0.032	0.0307
genotype	2.1	0.066	0.0083
Al:trait	2.6	0.08	0.0012
Al:genotype	2.1	0.127	0.001

**Pairwise comparison**	**F**	**R^2^**	***P***

0Al-T^b^ vs 0Al-S^c^	1.7	0.145	0.009
0.2Al-T^b^ vs 0.2Al-S^c^	1.5	0.132	0.016
0.4Al-T^b^ vs 0.4Al-S^c^	2.4	0.196	0.009

*^*a*^Sensitive versus tolerant, ^*b*^Al-tolerant soybean genotypes, ^*c*^Al-sensitive soybean genotypes.*

**FIGURE 3:**
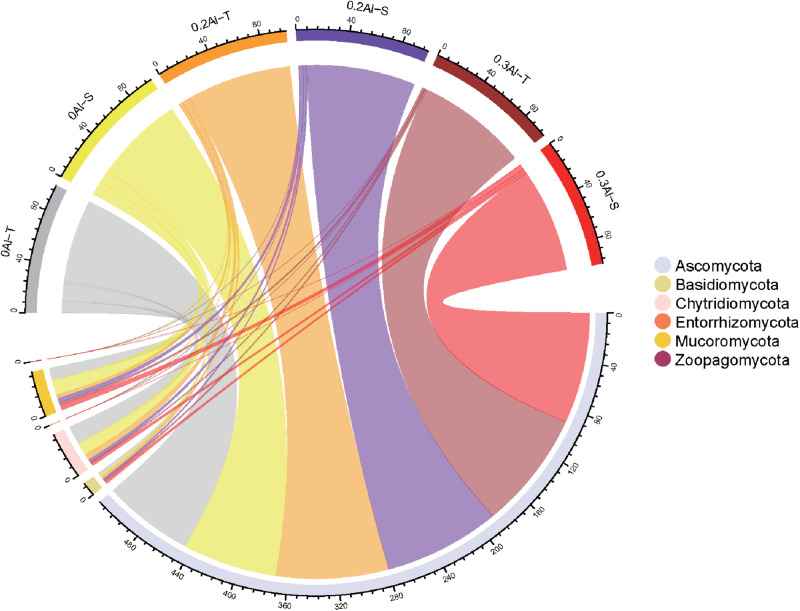
The relative abundance of rhizosphere fungi at the phylum level was detected in all soil samples. The color of the top half of the outer ring represents the corresponding soil sample group. The lower half of the outer ring is colored to represents different fungi at the phylum level. The thickness of lines was proportional to the relative abundance of rhizosphere fungi at the phylum level. Al-T: Al-tolerant soybean genotypes; Al-S: Al-sensitive soybean genotypes.

Eight fungal phyla were identified in the dataset. The community was dominated by Ascomycota (mean relative abundance of 86.29 ± 10.94%), followed by Mucoromycota (5.88 ± 4.63%), Chytridiomycota (6.0 ± 8.3%), Basidiomycota (1.78 ± 1.3%), Zoopagomycota (0.04 ± 0.07%) and Entorrhizomycota (0.01 ± 0.02%) (Figure 3). For these six fungal phyla, no significant differences in relative abundance were observed between Al-T and Al-S genotypes at the different Al concentrations.

**FIGURE 4:**
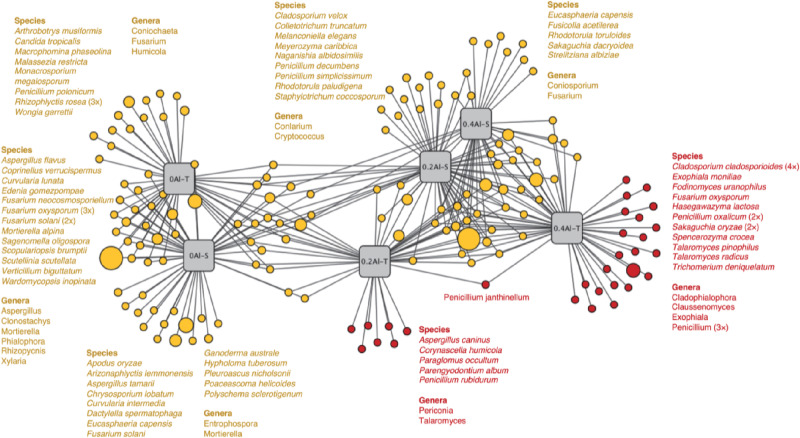
Bipartite association network showing positive associations between the treatment groups and the 163 significantly (*q* < 0.1) associated OTUs. Node sizes represent relative abundance of the OTUs. Edges represent the associations of individual OTUs with the treatments. The network structure was generated using the edge-weighted (association strength) Fruchterman-Reingold algorithm such that OTUs with similar associations and treatments with similar structure are clustered. Al-T: Al-tolerant soybean genotypes; Al-S: Al-sensitive soybean genotypes.

**FIGURE 5:**
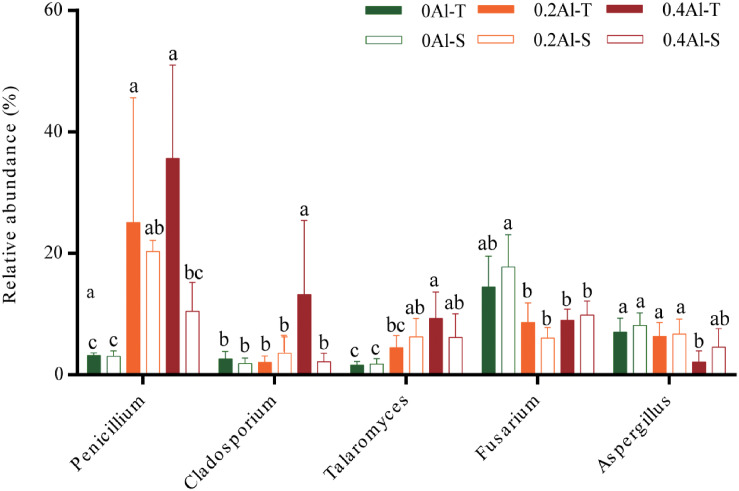
The relative abundance of the genera that associated to Al-tolerant soybean genotype. Al-T: Al-tolerant soybean genotypes; Al-S: Al-sensitive soybean genotypes.

Fungal taxa that significantly changed between the sensitive and tolerant genotypes across the different Al concentrations were identified using indicator species analysis and visualized using a bipartite association network (Figure 4). A total of 163 out of 618 OTUs (26%) representing 61% of the sequences were significantly (*q* < 0.1) associated to one or more treatment groups (Figure 4 and Supplementary Table S2). Among these OTUs, 74 were most strongly associated with only one treatment (Figure 4), confirming the basic distinctness of the communities in all six treatments. A total of 10 and seven OTUs were most strongly associated with the Al-S genotypes under low and high Al concentrations, respectively, whereas seven and 22 OTUs were associated with the Al-T genotypes under the low and high Al concentrations, respectively (Figure 4). Only OTU58 (*Penicillium janthinellum*) was associated with cross-combination of the Al-T genotype under the low and high Al concentrations (Figure 4). Abundant genera (>1%) that associated with Al-T genotypes included *Penicillium*, *Cladosporium* and *Talaromyces* and increased with increasing Al concentration (Figure 5). Conversely, *Aspergillus* and *Fusarium* decreased with increasing Al concentration, while revealing no significant difference between the tolerant and sensitive genotypes (Figure 5).

### Networks Analysis of Rhizosphere Fungal Communities

**FIGURE 6:**
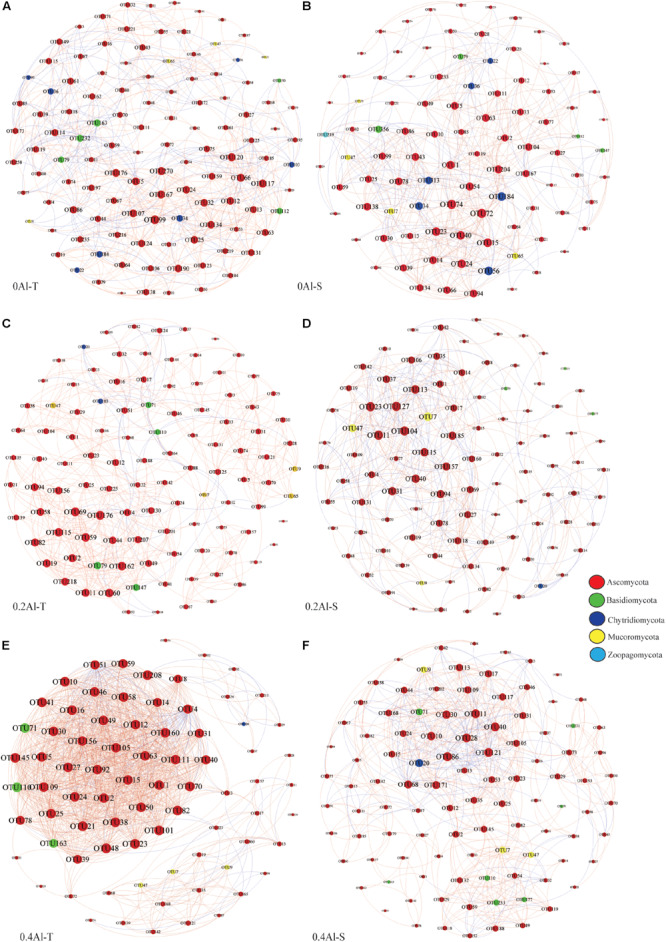
Co-occurrence network of the rhizosphere fungal community for different Al concentrations and soybean genotypes, i.e., 0 Al-T **(A)**, 0 Al-S **(B)**, 0.2 Al-T **(C)**, 0.2 Al-S **(D)**, 0.4 Al-T **(E)** and 0.4 Al-S **(F)**. Nodes represent OTUs colored-coded by phyla and scaled proportional to the number of connections (node degree). Connections were drawn at *r* > 0.8 (positive correlations, red) or *r* < –0.8 (negative correlations, blue) and *P* < 0.05. Al-T: Al-tolerant soybean genotypes; Al-S: Al-sensitive soybean genotypes.

Fungal co-occurrence network structure of the two genotypes changed with increasing Al concentration. At zero Al addition, network structure in term of positive correlation edges, average node degree, graph density and modularity were similar between the two genotypes (Figures 6A,B and Table 2). At low Al concentration, number of negative correlation edges and modularity was higher for Al-S than Al-T genotypes, while positive correlation edges were more frequent for Al-T (Figures 6C,D and Table 2). At high Al concentration, the Al-T genotypes had higher average node degree, as well as positive correlations when compared to the Al-S genotypes, but the average path length and modularity were lower (Figures 6E,F and Table 2). Hub OTUs were identified by calculating node degree, closeness centrality and betweenness centrality for all nodes in the network (Table 3). For example, OTU48 (*Aspergillus*) and OTU208 (*Talaromyces*) were identified as central hubs for the Al-T genotype under high Al concentration (Table 3).

**TABLE 2:** Topological characteristics of rhizosphere fungal networks across the different Al concentrations and soybean genotypes.

**Network metrics**	**0Al-T^a^**	**0Al-S^b^**	**0.2Al-T^a^**	**0.2Al-S^b^**	**0.4Al-T^a^**	**0.4Al-S^b^**
Number of nodes	116	95	108	101	78	103
Number of edges	812	582	662	567	1138	732
Number of positive correlations	551	439	600	374	1026	541
Number of negative correlations	261	143	62	193	112	191
Network diameter	6	10	7	8	9	8
Interconnecting piece	2	1	3	2	3	3
Graph density	0.122	0.13	0.12	0.11	0.37	0.13
Average node degree (avgK)	14	12.25	12.26	11.23	29.17	14.21
Average weighted node degree	4.91	5.91	9.74	3.52	39.31	6.96
Average clustering coefficient (avgCC)	0.62	0.66	0.64	0.67	0.86	0.68
Average path length (APL)	3.01	3.19	3.17	3.50	2.44	3.10
Modularity (M)	1.48	1.02	0.67	2.96	0.18	1.18

*^*a*^Al-tolerant soybean genotypes. ^*b*^Al-sensitive soybean genotypes.*

**TABLE 3:** Topological characteristics of hubs observed in rhizosphere soils across the different Al concentrations and soybean genotypes.

**Genotype**	**Phylum**	**Class**	**Order**	**Family**	**Genus**	**Species**	**OTU**	**Degree**	**Closeness centrality**	**Betweenness centrality**
0Al-T^a^	Ascomycota	Sordariomycetes	Microascales	Microascaceae	*Cephalotrichum*	*Unclassified Cephalotrichum*	OTU5	23	0.40	316.14
	Ascomycota	Sordariomycetes	Trichosphaeriales	Trichosphaeriaceae	*Nigrospora*	*Nigrospora_oryzae*	OTU176	23	0.40	316.14
0Al-S^b^	Ascomycota	Sordariomycetes	Hypocreales	Nectriaceae	*Fusarium*	*Fusarium oxysporum*	OTU72	24	0.41	193.58
	Ascomycota	Sordariomycetes	Glomerellales	Glomerellaceae	*Colletotrichum*	*Colletotrichum chlorophyti*	OTU74	24	0.41	193.58
0.2Al-T^a^	Ascomycota	Sordariomycetes	Glomerellales	Plectosphaerellaceae	*Plectosphaerella*	*Unclassified Plectosphaerella*	OTU69	24	0.36	135.15
	Ascomycota	Sordariomycetes	Trichosphaeriales	Trichosphaeriaceae	*Nigrospora*	*Nigrospora oryzae*	OTU176	24	0.36	135.15
0.2Al-S^b^	Ascomycota	Sordariomycetes	Sordariales	Chaetomiaceae	*Corynascella*	*Corynascella humicola*	OTU157	22	0.38	458.28
	Ascomycota	Dothideomycetes	Capnodiales	Cladosporiaceae	*Cladosporium*	*Cladosporium cladosporioides*	OTU11	26	0.35	129.68
0.4Al-T^a^	Ascomycota	Eurotiomycetes	Eurotiales	Aspergillaceae	*Aspergillus*	*Aspergillus niger*	OTU48	46	0.56	155.33
	Ascomycota	Eurotiomycetes	Eurotiales	Trichocomaceae	*Talaromyces*	*Talaromyces pinophilus*	OTU208	45	0.49	261.89
0.4Al-S^b^	Ascomycota	Sordariomycetes	Hypocreales	Nectriaceae	*Fusarium*	*Unclassified Fusarium*	OTU86	32	0.43	243.72
	Ascomycota	Dothideomycetes	Capnodiales	Cladosporiaceae	*Cladosporium*	*Cladosporium cladosporioides*	OTU11	31	0.43	220.02

*^*a*^Al-tolerant soybean genotypes. ^*b*^Al-sensitive soybean genotypes.*

### Soil Characteristics Were Linked to Changes of Fungal Communities

CCA and mantel testing were performed to identify correlations between soil characteristics and fungal community structure, indicating that fungal community structures were significantly correlated with specific soil characteristics such as available phosphorus, exchangeable H^+^, exchangeable Al^3+^, NH_4_^+^-N, NO_3_^–^-N, and pH (Supplementary Figure S2 and Table 4). In addition, TC also correlated with fungal community structure of the Al-T genotypes, which shifted with increase of Al concentration along CCA1 (Supplementaty Figure S2A).

**TABLE 4:** Analysis of the correlation (*r*) and significance (*P*) values between environmental factors and fungal communities by Mantel test. Al-T: Al-tolerant soybean genotype; Al-S: Al-sensitive soybean genotype.

	**r (Al-T^a^)**	***P* (Al-T^a^)**	**r (Al-S^b^)**	***P* (Al-S^b^)**	**r (High-Al)**	***P* (High-Al)**
pH	0.499	**0.004**	0.624	**0.004**	–0.031	0.582
Exchangeable H^+^	0.554	**0.004**	0.356	**0.010**	–0.033	0.636
Exchangeable Al^3+^	0.611	**0.004**	0.359	**0.012**	–0.008	0.636
TC	0.309	**0.013**	–0.059	0.759	0.156	0.272
TN	–0.114	0.821	–0.112	0.288	0.043	0.534
C:N	–0.026	0.636	0.094	0.337	0.261	0.209
TK	–0.018	0.636	–0.058	0.710	0.216	0.287
TP	–0.018	0.636	–0.096	0.821	–0.064	0.636
AP	0.418	**0.004**	0.734	**0.004**	0.314	0.240
NH_4_^+^-N	0.300	**0.015**	0.472	**0.004**	–0.033	0.636
NO_3_^–^-N	0.532	**0.004**	0.589	**0.004**	–0.002	0.2474

*^*a*^Al-tolerant soybean genotypes. ^*b*^Al-sensitive soybean genotypes.*

## Discussion

The aim of this study was to reveal the effects of Al stress on the rhizosphere fungal community structure of aluminum sensitive (Al-S) and tolerant (Al-T) soybean genotypes. The Al tolerant genotype harbored more abundant and structurally different fungal communities when compared to the sensitive genotype (Figures 1, 2). This finding supports the hypothesis that the plant, besides directly secreting more organic acid to chelate Al, might also recruit specific fungal species to the rhizosphere that themselves secret organic acids for Al detoxification (Kochian, 1995; Ma et al., 1997; Yang et al., 2012b). Thus, different soybean genotypes secrete different amounts and types of organic acids that cause different response to Al toxicity (Ryan et al., 2001; Kochian et al., 2015). However, fungal alpha-diversity showed no difference among the treatments, which is in contrast with our first hypothesis. This result indicated that fungal diversity was stable in the rhizosphere and was not affected by Al stress and soybean genotypes in this study. Notably, Al stress tended to increase difference in fungal community composition between the tolerant and sensitive genotypes, which is in accordance to what has been observed for bacteria (Lian et al., 2019).

Al stress reduced fungal abundance indicating that Al inhibited the growth of soil fungi (He et al., 2012). Moreover, Al stress altered the rhizosphere fungal community composition of both genotypes (Figure 2), which is consistent with previous studies suggested that Al affects fungal community structure (Vosátka et al., 1999; He et al., 2012). However, the observation that fungal community structure differed between the genotypes even without Al stress is in contrast with the study by Wang et al. (2009) reporting that fungal communities of three soybean genotypes were not different at the same growth stage. This discrepancy could be explained by the fact that different genotypes were investigated, different soil types were tested, and fungal communities were assessed using lower-resolution molecular methods.

Several studies have shown that Al tolerant genotypes can secrete more organic acids to chelate more Al ions (Kochian, 1995; Ma et al., 1997; Innes et al., 2004; Yang et al., 2012b). Based on the indicator species analysis, we have revealed that certain fungal genera significantly associated with the tolerant genotypes at high Al concentration. The comparisons among the treatments has identified several fungal taxa that have increased in relative abundance in the rhizosphere of the tolerant plant, including *Penicillium*, *Cladosporium* and *Talaromyces*. *Penicillium* has previously been shown to be highly tolerant to Al stress and therefore reduce Al toxicity by secreting organic acids and increasing soil pH (Zhang et al., 2002). Previous study has also reported that *Penicillium* can promote plant growth via increasing nutrient status of plants (Vessey and Heisinger, 2001). *Cladosporium* has been shown tolerant toward heavy metals, and could transfer phosphorus to the plant and promote plant growth under phosphorus deficiency, thereby cope with the Al toxicity (Bewley, 1980; Shao and Sun, 2007; Hiruma et al., 2016). *Talaromyces*, which is close to *Penicillium* and has initially been described as a sexual state of *Penicillium* species, are also known to be tolerant toward heavy metals (Yilmaz et al., 2014; Nam et al., 2019). Thus, the tolerance of soybean to Al toxicity may be closely related to the presence of these species.

A considerably large fraction of the community, representing 26% of the OTUs, responded significantly to Al addition and plant genotype (Figure 4). It has been suggested that aluminum contamination and soybean genotypes can affect entire clades of the rhizosphere microbial community structure by changing fundamental factors such as nutrition availability (Xu et al., 2009; Yang et al., 2012a). It is worth noting that some OTUs, e.g., OTU490 (*Penicillium decumbens*) and OUT 240 (*Penicillium simplicissimum*) associated with Al-S genotype under low Al concentration were affiliated to the genus *Penicillium*. Considering that this genus was significantly increased with Al stress and showed a higher relative abundance under the tolerant genotypes, it might contribute to Al tolerance with both sensitive and tolerant genotypes, and this contribution might be different for the two genotype groups (Figure 4).

Soil chemical properties, such as available phosphorus, NH_4_^+^-N, NO_3_^–^-N, exchangeable H^+^, exchangeable Al^3+^, and pH, were significantly correlated with shifts in fungal community structure of both genotypes (Table 4 and Supplementary Figure S2), and all these chemical properties were significantly associated with changing Al concentrations (Supplementary Table S1). These results suggested that the impacts of Al stress on the fungal communities might be directly linked to the alteration of soil chemical properties and vice versa.

Co-occurrence networks showed substantial structural differences between the Al tolerant and sensitive genotypes at both low and high Al concentrations. At the high Al concentrations, the fungal network of the tolerant genotypes revealed more negative correlations and lower modularity, which could be interpreted as representing increased inter-species competition according to network theory (Saavedra et al., 2011; Fan et al., 2018). Moreover, most fungal OTUs in Al-T genotypes are connected by positive links are considered to be unstable; in such a network, fungal OTUs may generated co-fluctuations and positive feedback along with environmental changes (Coyte et al., 2015; Vries et al., 2018). Besides, fungal hubs might also play an important role in mediating Al toxicity. For example, potential hub OTU48 was assigned to *Aspergillus*, which can produce organic acids that might alleviate Al toxicity by forming complexes with Al (Kawai et al., 2000).

In conclusion, aluminum stress had no effect on fungal diversity, but increased differences in fungal community structure between the sensitive and tolerant genotypes with increasing aluminum concentrations. Fungal genera such *Penicillium*, *Cladosporium*, and *Talaromyces* increased with increasing Al concentration and were enriched under the tolerant genotypes at high Al concentration. A more complex structure in fungal co-occurrence networks was found for the tolerant genotypes at high Al concentrations. However, to what extent these “enriched” fungal taxa have an impact on Al detoxification is not yet known and subject to future, more mechanistic experiments. These experiments also need to be carried out in different soil types and under different climatic conditions in order to evaluate the universality of the findings. This study highlights the possibility that rhizosphere fungi involved in Al detoxification can be used as breeding target.

## Data Availability Statement

The datasets generated for this study can be found in NCBI Bioproject, under accession number PRJNA561469.

## Author Contributions

TL and HN designed the research. TL, QS, YL, AS, and ZC performed the research. QS, TL, and MH analyzed the data and wrote the manuscript. All authors have read and approved the manuscript as submitted.

## Conflict of Interest

The authors declare that the research was conducted in the absence of any commercial or financial relationships that could be construed as a potential conflict of interest.

## Footnotes

1 http://unite.ut.ee
