# Supplementary material for: Rhizosphere Soil Fungal Communities of Aluminum-Tolerant and -Sensitive Soybean Genotypes Respond Differently to Aluminum Stress in an Acid Soil
Source: Front Microbiol. 2020 May 28;11:1177. doi: 10.3389/fmicb.2020.01177 (PMC7270577; doi:10.3389/fmicb.2020.01177)
Supplement: Supplementary file 1 [file Data_Sheet_1.docx]

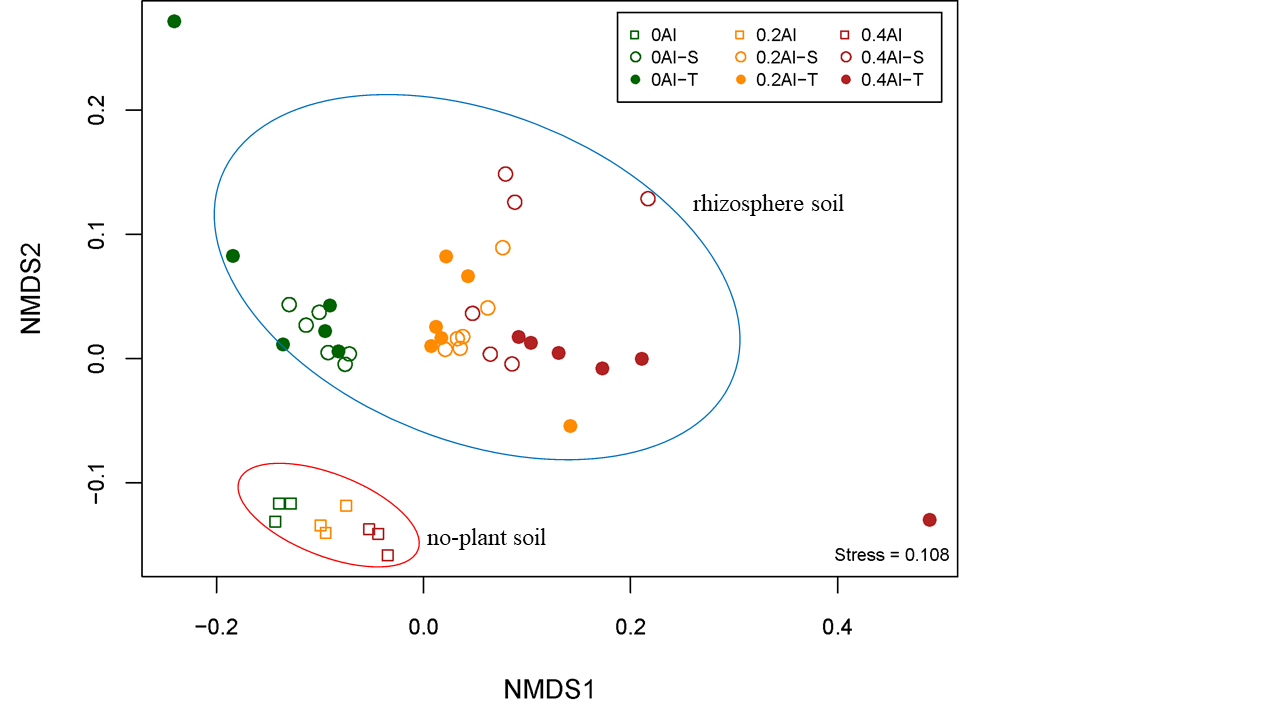


Fig. S1 Non-metric multidimensional scale (NMDS) based on Bray-Curtis dissimilarities showing differences in the no-plant soil and rhizosphere fungal community structures. Al-T: Al-tolerant soybean genotype; Al-S: Al-sensitive soybean genotype.

**
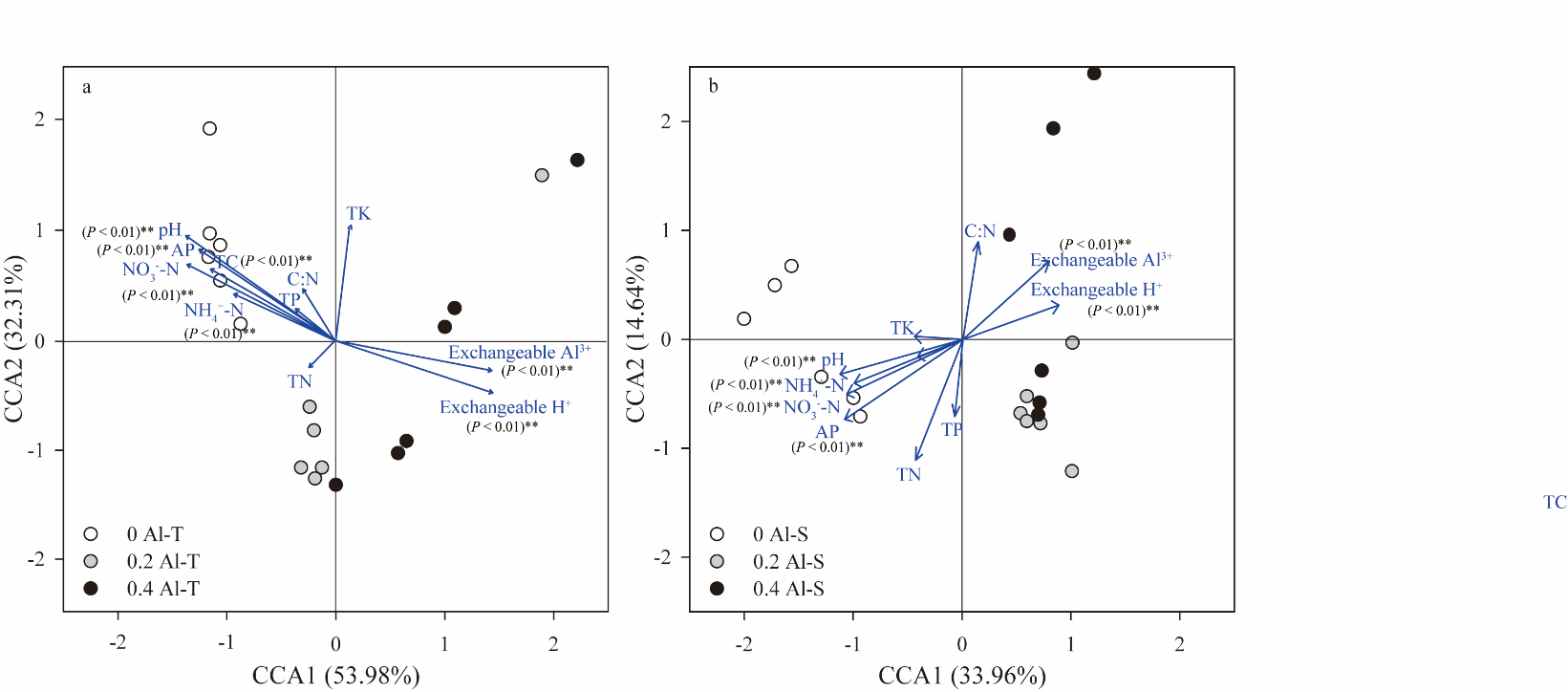
**

**Fig.** **S2** Canonical correspondence analysis (CCA) constraining rhizosphere fungal community structure by chemical soil properties across Al-T samples (a), Al-S samples (b). Arrows indicate the direction and magnitude of environmental parameters associated with bacterial community structure. Relationships between community ordinations scores and soil property vectors were examined by Mantel testing. pH, soil pH; TC, total carbon; TN, total nitrogen; C:N, ratio of TC to TN; TP, total phosphorus; AP, available phosphorus; NH_4_^+^-N, ammonium nitrogen; NO_3_^-^-N, nitrate nitrogen; Al-T: Al-tolerant soybean genotype; Al-S: Al-sensitive soybean genotype.
